# Effects of *Annona muricata* Extract on Trypsin, Cathepsin B and Collagenase Activities and Textural Changes in Chilled *Macrobrachium rosenbergii*

**DOI:** 10.3390/foods12091887

**Published:** 2023-05-04

**Authors:** Amalina Ibrahim, Kamariah Bakar, Jamilah Bakar, Nilesh Prakash Nirmal, Mhd Ikhwanuddin, Nurul Ulfah Karim

**Affiliations:** 1 Higher Institution Center of Excellence (HICoE), Institute of Tropical Aquaculture and Fisheries, Universiti Malaysia Terengganu, Kuala Nerus 21030, Malaysia; 2 Institute of Biotechnology Marine, Universiti Malaysia Terengganu, Kuala Nerus 21030, Malaysia; 3 Department of Food Technology, Faculty of Food Science and Technology, Universiti Putra Malaysia, Serdang 43400, Malaysia; 4 Institute of Nutrition, Mahidol University, 999 Phutthamonthon 4 Road, Nakhon Pathom 73170, Thailand

**Keywords:** *Macrobrachium rosenbergii*, texture, trypsin, cathepsin B, collagenase, quality, *Annona muricata* leaves

## Abstract

Texture is an important sensory attribute for overall quality and consumer acceptance of prawns. However, texture is affected during cold storage due to the proteolytic activity of endogenous proteases, resulting in poor quality and a short shelf life. The objective of this study is to determine the inhibitory effects of *Annona muricata* leaves extract (AMLE) (0, 3, 10 and 20%) on the trypsin, cathepsin B and collagenase activities extracted from the cephalothorax of *Macrobrachium rosenbergii*. In addition, the textural changes in *M. rosenbergii* during 20 days of cold storage (4 °C) were also determined. *M. rosenbergii* were soaked in four different treatments: 0, 3, 10 and 20% AMLE and 1.25% sodium metabisulphate for 10 min at 4 °C. Protease activity was significantly (*p <* 0.05) reduced at 10 and 20% AMLE. Similarly, cathepsin B showed a significant (*p <* 0.05) low after treatment at 20% AMLE. The maximum inhibitory activity of trypsin was achieved at 20% AMLE and the standard inhibitor (Tosyl-L-lysyl-chloromethane hydrochloride (TLCK)) compared to the control. Whereas, the lowest collagenase activity was obtained at 20% AMLE compared to the control. These inhibitory effects further maintain the firmness of *M. rosenbergii* coated with 20% AMLE up to the eighth day of storage when compared to the control. Meanwhile, the highest penetration work was found in the *M. rosenbergii* coated with 20% AMLE at the twentieth day of storage. In conclusion, treatment at 20% AMLE could be used as a natural preservative to inhibit protease, trypsin and collagenase activity of *M. rosenbergii* and thus can maintain firmness for up to 8 days of storage.

## 1. Introduction

The giant freshwater prawn *Macrobrachium rosenbergii* is an important aquaculture species and has high economic value. This aquaculture prawn species is native to the South Asian region and is extensively cultured in many countries such as Malaysia, Bangladesh, Thailand, Vietnam, China and India [1]. From 2015 to 2019, the gross production of *M. rosenbergii* in Malaysia was around 200 tonnes per year, and this production is expected to increase in the coming years to meet the global demand [2,3,4,5,6]. However, this high-value species has a limited shelf life of up to 3–4 days due to the rapid degradation of texture during storage [7]. Initially, the texture of *M. rosenbergii* is relatively firm; however, during extended storage at low temperatures, the muscle slowly degrades and becomes mushy, a phenomenon known as textural softening [8]. Textural softening is the result of endogenous protease activity in the hepatopancreas and muscles of the prawn [9].

Proteases are diverse group of enzymes that hydrolyze peptide bonds in proteins. Serine proteases of *M. rosenbergii*, mainly trypsin-like or chymotrypsin-like from the hepatopancreas, degrade the natural actomyosin and pepsin-soluble collagen from the muscle [10]. Trypsin-like collagenase and trypsin from the hepatopancreas showed collagenolytic activity towards pepsin-soluble collagen and degraded type 1 collagen from the muscle of *M. rosenbergii* [11]. The degradation of collagen and actomyosin, the contractile protein consisting of actin and myosin, from muscle tissue has contributed to the significant textural softening of *M. rosenbergii* during post-mortem storage [12]. Similarly, in the post-rigor phase, autolytic degradation causes a rupture in the lysosome and cathepsin are released from the lysosome into the cytosol [13]. Cathepsin B hydrolyzes myofibrillar proteins into smaller polypeptides chains [14,15]. The cathepsin was reported to decrease the shear force of grass carp (*Ctenopharyngodon idella*) fillets by degrading the muscle texture during post-mortem storage for 21 days [16].

Activity of collagenase from the hepatopancreas of *M. rosenbergii* is 15 to 40 times more active in degrading protein than trypsin [17]. Collagenase breaks down the collagen in the extracellular matrix of connective tissue, resulting in the loss of integrity of the muscle structure [18]. Activity of collagenase has also resulted in low shear force in muscle texture [19]. Collagenase binds and cleaves the triple helix at the Gly-Leu or Gly-Ile site located ¾ from the N-terminal end [20]. The muscle structure of *M. rosenbergii* degrades due to the activity of proteases, trypsin, cathepsin B and collagenase. The degradation causes a softening of the texture in the prawn. Textural softening is usually noticeable after 4 days of storage at the first abdomen, which later spreads to the other sections towards the tail and causes loosening and flaking in the cooked meat [21,22].

Thus, instrumental texture analysis can be used to determine the changes in texture of *M. rosenbergii*. The penetration test gives information about important mechanical parameters such as the force needed to deform the meat (firmness) and the work needed to penetrate the skin layer (penetration work) [23]. As the storage time increases, the values of firmness and penetration work change accordingly [24]. Softening of texture results in a low firmness value due to the degradation of muscle texture by the activity of proteases [25]. The penetration work increases as the skin stiffness increases during post-mortem storage [26]. Both firmness and penetration work provide valuable information on the textural changes in *M. rosenbergii* muscle during post-mortem storage.

Textural softening is associated with meat quality degradation and shelf-life reduction that can lead to reducing the market value of the prawns. Therefore, it is important to control the textural properties of prawns. The most effective way to control textural properties is by inhibiting or inactivating the endogenous protease using protease inhibitors [27]. Protease inhibitors interfere with the breakdown of peptide bonds during protein hydrolysis and inhibit proteolysis of the muscle [28]. Protease inhibitors work by reacting either with a substrate, enzyme or the final product in the reaction [29].

*Annona muricata*, commonly known as soursop, is an evergreen tree native to tropical regions around the world. Leaves extract of *A. muricata* contains acetogenins (anticancer compounds) such as 12, 15-cis-squamostatin-A, isodesacetyluvaricin, squamostatin-A, squamocin and bullatacin [30]. Additionally, leaves extract of *A. muricata* is reported to exhibit antioxidant, anti-inflammatory and antidiabetic activities [31,32,33]. Currently, there is no report on the effects of *A. muricata* leaves extracts against proteases activity and textural changes in *M. rosenbergii* during cold storage. A previous study by Ibrahim et al. [34] stated that the *A. muricata* leaves extract effectively inhibits the PPO enzyme, which is responsible for the melanosis phenomenon in *M. rosenbergii* muscles. Apart from melanosis, mushiness is another deteriorative reaction causing the unacceptability of prawns during storage. Mushiness or texture softening are caused by the degradation of the edible portion due to activity of proteolytic enzymes. The autolysis of the cephalothorax, where the hepatopancreas and other internal organs are located, releases the active proteinases such as trypsin and collagenase, which are capable of degrading the native collagen under physiological conditions into the muscle. Therefore, a study on the inhibitory effects of proteases, including trypsin, cathepsin B and collagenase, may benefit in keeping the freshness and appearance of *M. rosenbergii* during storage.

Therefore, the objectives of this study are to determine the inhibition effect of the AMLE on the activity of proteases, trypsin, cathepsin B and collagenase extracted from the cephalothorax of *M. rosenbergii* and determine the textural changes in *M. rosenbergii* treated with various concentrations of AMLE during 20 days of cold storage (4 °C).

## 2. Materials and Methods

### 2.1. Chemicals

Sodium phosphate monobasic, sodium phosphate dibasic and sodium chloride were procured from Merck (Japan). Sodium metabisulfite was purchased from Fisher Scientific (UK). Hydrochloric acid was obtained from Merck (Austria). Bovine serum albumin and Bradford reagent were procured from Sigma-Aldrich (USA).

### 2.2. Macrobrachium Rosenbergii Preparation

Commercial-sized (20–25 pieces per kg) giant freshwater prawns (*M. rosenbergii*) were purchased from a local hatchery and brought to the laboratory in an ice container. Upon arrival, prawns were acclimatized in a tank supplied with oxygen for 48 h. The prawns were separated in two groups with 200 individuals in each group: (i) use of cephalothoraxes for endogenous protease extraction, and (ii) whole prawn for *A. muricata* leaves extract (AMLE) treatment. The cephalothoraxes were separated and carefully poured with liquid nitrogen and ground to powder form prior experiment.

### 2.3. Annona Muricata Preparation and Extraction

*A. muricata* leaves were cleaned and shade-dried under natural air flow at room temperature (25 °C) for 7 days (moisture content 16.51 ± 0.03%). The leaves were grounded using a Waring blender (Waring Commercial, Malaysia) and sieved with a 500 µm mesh size. The leaves extract was obtained by mixing 30 g of powder with 200 mL methanol-distilled water solvent (6:4, *v*/*v*) [34]. The mixture was shaken for 24 h with a Stuart Orbital Shaker (Fisher Scientific, Malaysia), followed by filtration with Whatman filter paper number 1. Mixture was dried using rotary evaporator R-210 (Buchi, Switzerland) to remove excessive solvent, and the obtained extract was kept in container.

### 2.4. Determination of Total Phenolic and Flavonoid Content

Total phenol content (TPC) was determined using Folin–Ciocalteu method [35]. An aliquot of 1 mL sample was added into distilled water and Folin–Ciocalteu reagent (1:1). The mixture was added to 1.5 mL of 20% sodium carbonate after left to stand for five minutes. The mixture was made up to 10 mL with distilled water and incubated for two hours at room temperature. The absorbance reading was taken with UV-1800 spectrophotometer (Shimadzu, Malaysia) at 750 nm. Gallic acid was used as a standard, and the result was expressed as mg of gallic acid equivalent (GAE) g^−1^ dry mass.

Total flavonoid content (TFC) was determined according to Kametaker et al. 2014 [35]. An aliquot of 1 mL sample was added into 4 mL of distilled water and 0.3 mL of 5% sodium nitrite. The mixture was added with 0.3 mL of 10% aluminum chloride after left to stand for 5 min. An aliquot of 2 mL 1 M sodium hydroxide was added after 1 min before the volume was made up to 10 mL with distilled water. The absorbance reading was taken using UV-1800 spectrophotometer (Shimadzu, Malaysia) at 510 nm. Quercetin was used as standard, and the result was expressed as mg of quercetin equivalent (QE) g^−1^ dry weight.

### 2.5. Protease Activity

#### 2.5.1. Extraction of Crude Protease from Cephalothorax Powder

The crude protease extract was prepared using the method of Sriket et al. 2011b [12]. In 100 mL of extracting buffer (0.01 M sodium phosphate buffer, pH 7.6), 50 g of cephalothorax powder was dissolved and homogenized at 10,000 rpm for 2 min. The homogenate was stirred for 30 min and centrifuged using a refrigerated centrifuge (Hitachi, Japan) at 11,200× *g* for 30 min at 4 °C. The supernatant collected was referred to as ‘crude protease extract’.

#### 2.5.2. Determination of Total Protein

The total protein in the crude protease extract was determined using the Bradford assay with some modifications on the concentration of standard used [36]. A range of concentrations of bovine serum albumin (0.0–1.0 mg mL^−1^) was used as standards. An aliquot of 10 µL of sample was added into 200 µL of Bradford reagent, followed by incubation at room temperature for 10 min. The absorbance of the samples was recorded at 595 nm using a microplate reader (Thermo Fisher Scientific, Finland). The total protein was determined by comparing the absorbance reading with the standard curve plotted and expressed as mg mL^−1^.

### 2.6. Inhibitory Effect of Annona Muricata Leaves Extract (AMLE) on Protease Activity

The activity of crude protease extract was determined with the Protease Activity Assay Kit (Cayman Chemical, USA). The assay kit was developed based on a method by Twining, 1984 [37]. The assay utilized fluorescein isothiocyanate (FITC) as a fluorescein tag bound to casein substrate. As a background reading, 100 µL of buffer solution (25 mM Tris, pH 7.2, containing 150 mM sodium chloride) and 100 µL of casein solution (containing 80 µL of casein and 20 µL buffer) were added into a 96-well microplate. In enzyme control, 100 µL crude protease extract was added with 100 µL buffer solution.

An aliquot of 100 µL of crude protease extract was pipetted into a 96-well microplate. Next, 20 µL of AMLE (0, 3, 10 and 20%, respectively) was added. A total of 80 µL of casein was added to start the reaction. The mixture was incubated at room temperature for 20 min. The absorbance was determined using a SpectraMax iD3 Multi-mode microplate reader (Molecular Devices, USA) with 480 nm excitation wavelength and 516 nm emission wavelength. The result was expressed as protease activity in the unit of relative fluorescence unit (RFU) min^−1^ mg^−1^. The experiment was repeated in triplicate. Calculation of protease activity was carried out according to Haydar and Kubra, 2016; Ching et al. 2013 [38,39].

Protease activity (RFU min^−1^ mg^−1^)

$$\frac{Amount\;of\;activity\;unit\;(Enzyme\;unit\;(RFU)/Incubation\;time\left(min\right)\times Assay\;volume\left(mL\right)}{Total\;protein\;concentration\;({mg.mL}^{-1})}$$

### 2.7. Inhibitory Effect of AMLE on Trypsin-Like Activity

Trypsin activity was determined with the Trypsin Activity Colorimetric Assay Kit (Biovision Research Products, USA). Trypsin cleaved substrate to generate p-nitroaniline (p-NA) at 405 nm. The standard curve was prepared at range of 0–10 µL p-NA standard, and the volume was made up to 50 µL with Trypsin Assay Buffer. The concentration of p-NA standard was 0–20 nmol per well, accordingly. In background sample, 96 µL trypsin assay buffer was added into 5 µL trypsin substrate. Then, 5 µL of trypsin substrate was added, and the volume was made up to 101 µL with Trypsin Assay Buffer. In enzyme control, 50 µL of crude protease extract was added to 51 µL trypsin assay buffer in a 96-well microplate.

The assay was conducted as per protocol (Biovision Research Products, USA) with some modifications on addition of AMLE as an enzyme inhibitor. A total of 50 µL of crude protease extract was added into the 96-well microplate. An aliquot of 1 µL of AMLE (0, 3, 10 and 20%, respectively) was added into the 96-well microplate. Next, 45 µL of Trypsin Assay Buffer and 5 µL of trypsin substrate were added to initiate the reaction. The microplate was incubated at 25 °C in the dark. Tosyl-L-lysyl-chloromethane hydrochloride (TLCK) (20 mM) was used as a standard inhibitor. The absorbance reading was recorded at 405 nm using a SpectraMax iD3 Multi-mode microplate reader (Molecular Devices, USA) for 2 h. One unit (U) was defined as the amount of trypsin that cleaved the substrate, yielding 1.0 µmol of p-NA per minute at 25 °C. The trypsin activity was determined using the following formula:

$$\mathrm{Trypsin}\;\mathrm{activity}\;(µU\;{mL}^{-1})=\frac{B}{\left(T_{2}-T_{1}\right)V}\times \mathrm{sample}\;\mathrm{dilution}\;\mathrm{factor}$$

where:

B is the p-NA calculated from the standard curve (nmol);

*T*_2_ − *T*_1_ is the time of the first and second readings (min);

*V* is the sample volume added into the reaction well (mL).

### 2.8. Inhibitory Effect of AMLE on Cathepsin-B-Like Activity

Cathepsin B activity was determined with the Cathepsin B Activity Fluorometric Assay Kit (Biovision Research Products, USA). Cathepsin B in the sample cleaved cathepsin B (CB) substrate sequence RR labeled with amino-4-trifluoromethyl coumarin (AFC). The enzymatic reaction produced free AFC which was determined using a fluorescence microplate reader. In background sample, 50 µL of CB reaction buffer was added into 50 µL of 10 mM CB substrate Ac-RR-AFC, and the volume was made up to 104 µL by adding CB reaction buffer. In enzyme control, 52 µL crude protease extract was added with 52 µL CB reaction buffer in the 96-well microplate.

The assay was conducted by referring to the protocol (Biovision Research Products, USA) with some modifications on addition of AMLE as an enzyme inhibitor. Next, 50 µL of crude protease extract was added into 50 µL CB Cell Lysis Buffer. The mixture was incubated for 10 min before centrifuged (Hitachi, Japan) at 13,000 rpm for 5 min at 4 °C. Supernatant was collected and labeled as ‘cell lysate’. A total of 50 µL of cell lysate was pipetted into the 96-well microplate. An aliquot of 50 µL of CB reaction buffer and 2 µL of CB Substrate Ac-RR-AFC were added into the 96-well microplate. Next, 2 µL of AMLE (0, 3, 10 and 20%, respectively) was pipetted into the 96-well microplate. The plate was incubated at 37 °C for 1 h. The absorbance was determined using a SpectraMax iD3 Multi-mode microplate reader (Molecular Devices, USA) with 400 nm excitation wavelength and 505 nm emission wavelength. Cathepsin B inhibitor (1 mM) was used as a standard inhibitor. Experiment was conducted in triplicate. Calculation of cathepsin B activity was performed according to the studies by Haydar and Kubra, 2016; Ching et al. 2013 [35,36].

Cathepsin B activity (RFU min^−1^ mg^−1^):

$$\frac{Amount\;of\;activity\;unit\;[Enzyme\;unit\;(RFU)/\left(Incubation\;time\left(min\right)\right]\times Assay\;volume\;(mL)}{Total\;protein\;concentration\;({mg.mL}^{-1})}$$

### 2.9. Inhibitory Effect of AMLE on Collagenase-Like Activity

Collagenase activity was determined using Collagenase Activity Colorimetric Assay Kit (Biovision Research Products, USA). The kit used synthetic peptide, N-3-(2-Furyl) acryloyl-Leu-Gly-Pro-Ala (FALGPA), as a substrate that mimickedt the collagen structure. In background sample, 100 μL of Collagenase Assay Buffer was used as a reagent. Then, 40 µL of FALGPA substrate was added, and the volume was made up to 200 µL by adding collagenase assay buffer. The volume was made up to 200 μL by adding Collagenase Assay Buffer. In enzyme control, 100 µL crude protease extract was added into the 96-well microplate, followed by 100 µL collagenase assay buffer.

The assay was conducted with some modifications (Biovision Research Products, USA) on addition of AMLE as an enzyme inhibitor. An amount of 10 µL of crude protease extract was added to the 96-well microplate. Next, 2 µL AMLE (0, 3, 10 and 20%) was added to the 96-well microplate. The volume was made up to 100 µL with Collagenase Assay Buffer. A total of 40 µL of FALGPA substrate and 60 µL of Collagenase Assay Buffer were added into the microplate. The absorbance was immediately recorded using a spectrophotometer, SpectraMax iD3 Multi-mode microplate reader (Molecular Devices, USA) at 345 nm wavelength for 15 min. For a standard inhibitor, 10-Phenanthroline (1 M) was used. The absorbance reading (A_345nm1_ and A_345nm2_) was taken at two time points (T_1_ and T_2_). The experiment was conducted in triplicate. One unit (U) was defined as the amount of collagenase that cleaved the FALGPA substrate and produced 1.0 µmol of product per minute. The collagenase activity was calculated using the following formula:

Collagenase activity (U mL^−1^):

$$\frac{=(\frac{-\Delta A\left(345\;nm\right)}{\Delta T}Test—Reagent\;background)\times 0.2\times DF}{0.53\times V}$$

where:

ΔA_345 nm_ is the difference between two absorbance readings taken at two time points (T_2_ and T_1_);

ΔT is difference between T_2_ and T_1_ (min);

0.20 is reaction volume (mL);

DF is the dilution factor;

0.53 is the millimolar extinction coefficient of FALGPA;

V is the enzyme volume (mL).

### 2.10. Treatment of M. rosenbergii with AMLE

*M. rosenbergii* were subjected to the ice-killed method with prawn:ice ratio of 1:2 (*w*/*w*) before further experiment in conjunction to reduce nerve function and metabolic activity. They were then cleaned under cold water. Samples were randomly put into containers containing four different treatments; 3, 10 and 20% AMLE and 1.25% sodium metabisulphate (SMS) for 10 min at 4 °C. Controls were left without any treatments. All samples were frozen in a blast freezer at −18 °C (Irinox Blast Freezer, USA) for 5 min to rapidly freeze the coatings before being kept in polyethylene bags and stored in the cold storage at 4 °C for 20 days. Samples were thawed by placing on the bed of ice before taken on day 0 and thereafter every 4th day for textural analysis.

### 2.11. Instrumental Texture Analysis

The texture of *M. rosenbergii* was evaluated using a textural analyzer TAXT plus (Stable Micro Systems, UK) according to the method of Nunak and Schleining, 2011 [25]. All samples were subjected to the penetration test to obtain penetration work and firmness values. The instrument was operated using Exponent software and calibrated with a 5 kg load cell before any measurements of data were taken. The instrument was set up to a pre-test speed of 1.0 mm s^−1^, test speed of 0.10 mm s^−1^, post-test speed of 1.0 mm s^−1^ and trigger force of 5.0 g. The probe used was a cylindrical flat-ended P/3 3 mm diameter probe. To start the experiment, the raw prawn was beheaded and peeled off, and then it was put on the raised platform directly underneath the probe. The penetration test was carried out on the second segment of the prawn to obtain the best firmness and penetration work values. Data of firmness (N) and work of penetration (kg s) were collected using the Exponent software every 4 days for 20 days. The experiment was conducted in triplicate.

### 2.12. Statistical Analysis

All the analyses were performed in triplicate (*n* = 3), and the data were represented as mean ± standard error. The collected data were analyzed using the IBM SPSS Statistic Version 20 software (IBM Corporation, New York, NY, USA). The data were subjected to one-way ANOVA and post hoc test. In all analyses by ANOVA, the residuals were inspected for normality and homogeneity of variance across the treatments. Different superscripts (A, B, C, D, E) indicated significant differences (*p <* 0.05) among storage days. Meanwhile, different superscripts (a, b, c) indicated significant differences (*p <* 0.05) between treatments.

## 3. Results

### 3.1. Total Phenolic and Flavonoid Contents of AMLE

Total phenolic content (TPC) and total flavonoid content (TFC) in the AMLE were recorded at 93.90 ± 0.05 mg GAE g^−1^ and 53.38 ± 0.01 mg QE g^−1^, respectively.

### 3.2. Inhibitory Effects of AMLE on Protease Activities

#### 3.2.1. Protease Activity

**Figure 1:**
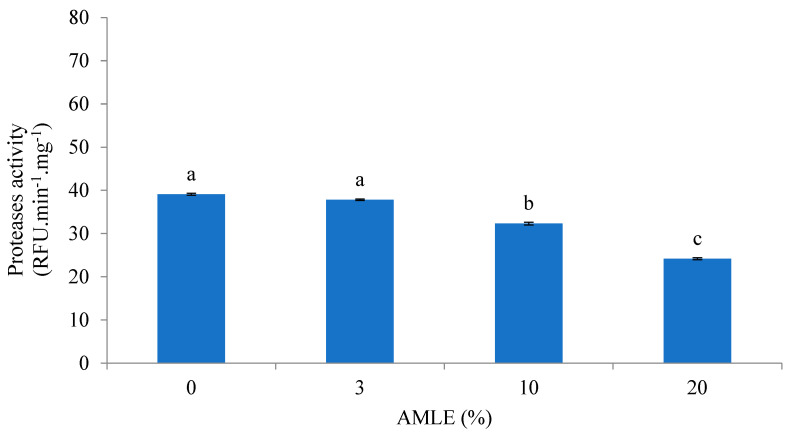
Inhibitory effect of AMLE against protease from hepatopancreas of *M. rosenbergii*. RFUmin^−1^ mg^−1^ was expressed in 10,000 display unit. Different superscripts (a, b, c) indicate significant differences (*p* < 0.05) between treatments 0–20% *A. muricata*).

The AMLE showed dose-dependent inhibitory activity against protease activity from *M. rosenbergii* carapaces. In general, 3% of the AMLE did not show any significant inhibitory effects compared to the control (*p >* 0.05). The significant protease inhibition was observed at the concentrations of 10 and 20% AMLE (*p <* 0.05) (Figure 1).

#### 3.2.2. Cathepsin B Activity

**Figure 2:**
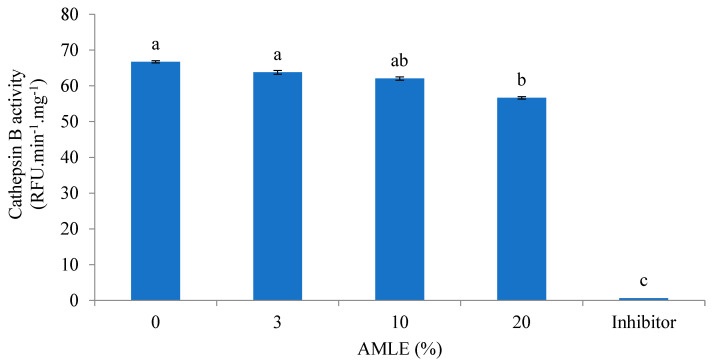
Inhibitory effect of AMLE against cathepsin-B-like activity from hepatopancreas of *M. rosenbergii*. RFUmin^−1^ mg^−1^ was expressed in 100,000 display unit. Different superscripts (a, b, c) indicate significant differences (*p* < 0.05) between treatments (0–20% *A. muricata*). Standard inhibitor: Cathepsin B inhibitor (CIB) (1 mM).

The inhibitory effect of the AMLE was evaluated against cathepsin-B-like activity (Figure 2). The AMLE at a 20% concentration significantly inhibited the cathepsin B activity. Meanwhile, the inhibitor showed significant (*p* < 0.05) reduced cathepsin B activity compared to other treatments.

#### 3.2.3. Trypsin Activity

**Figure 3:**
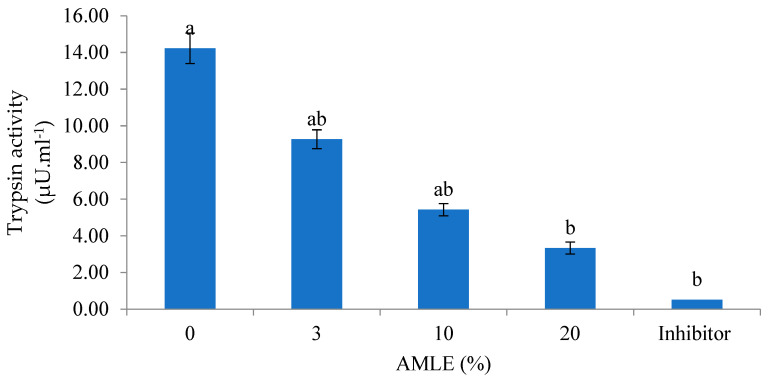
Inhibitory effect of AMLE against the trypsin-like activity from hepatopancreas of *M. rosenbergii*. Different superscripts (a, b) indicate significant differences (*p* < 0.05) between treatments (0–20% *A.muricata*). Standard inhibitor: Tosyl-L-lysyl-chloromethane hydrochloride (TLCK) (20 mM).

Trypsin is a serine protease and is known to digest into small fragments. Therefore, it was worth evaluating the inhibitory effect of the AMLE on the trypsin-like activity from the hepatopancreas of *M. rosenbergii*. The results showed that the AMLE reduced the trypsin-like activity at all tested concentrations (0–20%) (Figure 3). The inhibitory effect of the AMLE was dose-dependent. The maximum inhibitory activity was achieved at 20% AMLE and the standard inhibitor compared to the control (Figure 3).

#### 3.2.4. Collagenase Activity

**Figure 4:**
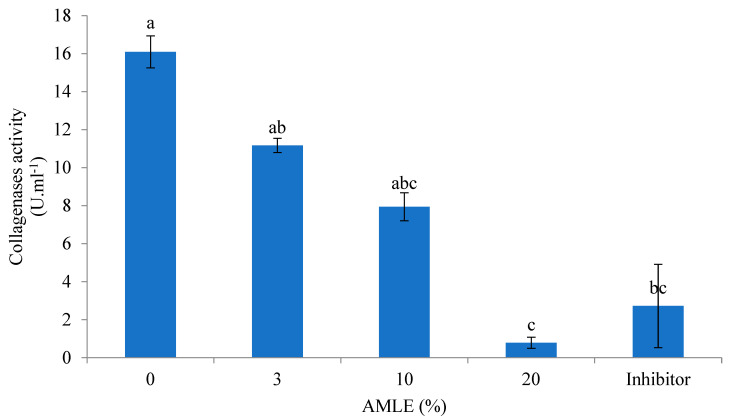
Inhibitory effect of AMLE against collagenase-like activity from hepatopancreas of *M. rosenbergii*. Different superscripts (a, b, c) indicate significant differences (*p* < 0.05) between treatments (0–20% *A. muricata*). Standard inhibitor: 10-Phenanthroline (1 M).

The AMLE at 20% concentration showed the lowest (*p* < 0.05) inhibitory effect when compared to the control (0% AMLE). Similarly, the standard inhibitor also showed an inhibitory effect compared to the control (Figure 4).

### 3.3. Textural Analyses of Macrobrachium Rosenbergii Treated with AMLE

#### 3.3.1. Firmness

**Figure 5:**
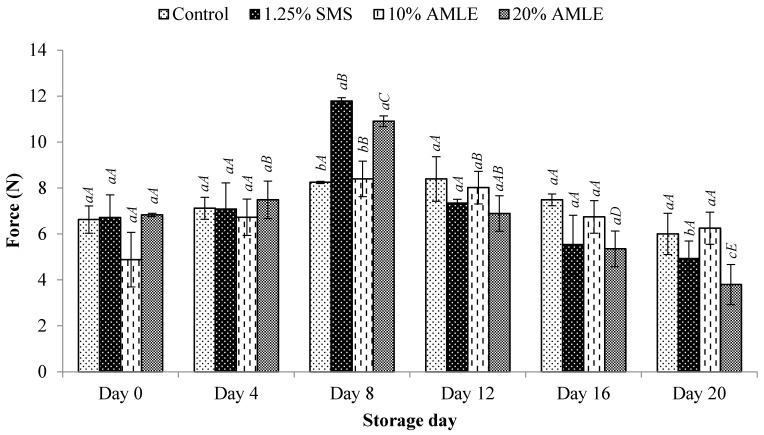
Firmness value of *M. rosenbergii* during 20 days of cold storage. Small letters (a, b, c) indicate significant differences between treatments on same storage day (*p* < 0.05). Capital letters (A, B, C) indicate significant differences between storage days for same treatment (*p* < 0.05).

The effects of the AMLE treatments on the firmness of *M. rosenbergii* are shown in Figure 5. In general, the firmness of the control showed no significant (*p* > 0.05) changes during 20 days of storage (Figure 5). A similar trend was observed with the samples treated with either SMS or AMLE. The highest firmness was observed in samples treated with SMS and 20% AMLE at 8 days of cold storage. Thereafter, a decrease in firmness was noted for both samples (*p* < 0.05). The firmness of shrimp coated with 10% AMLE was significantly (*p* < 0.05) higher on the 8th day of storage, compared to the shrimp coated with 10% AMLE on the 16th day of storage (Figure 5). However, the firmness of *M. rosenbergii* coated with 20% AMLE was recorded to give the highest value (10.907 ± 0.398 N) on day 8 before decreasing significantly (*p* < 0.01) to 5.349 ± 1.342 N on the 16th day of storage (Figure 5). A high firmness value is preferable during the prolonged storage time.

#### 3.3.2. Penetration Work

**Figure 6:**
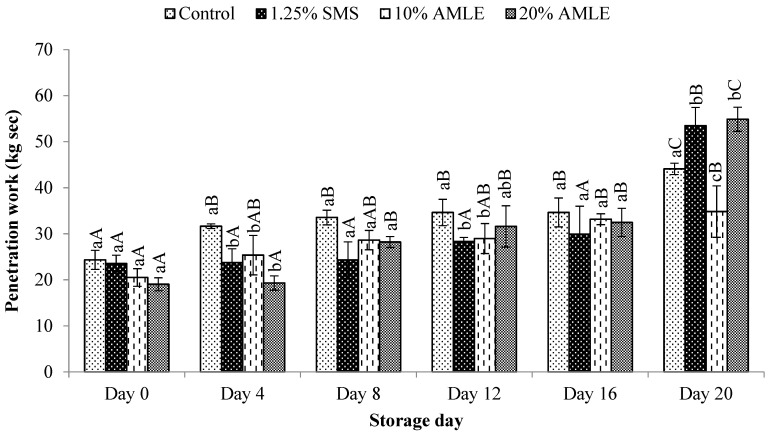
Effect of AMLE treatment at different concentrations on penetration work value of *M. rosenbergii* during 20 days of cold storage. Small letters (a, b, c) indicate significant differences between treatments in one day (*p* < 0.05). Capital letters (A, B, C) indicate significant differences between storage days in one treatment (*p* < 0.05).

The penetration work of *M. rosenbergii* treated with different concentrations of AMLE during 20 days of cold storage is presented in Figure 6. The penetration work for all samples showed a steady increase (*p* < 0.05) with prolonged storage duration (Figure 6). When compared with different AMLE concentrations, 10% AMLE treatment had lower penetration work at the end of the storage day compared to 20% AMLE. Surprisingly, prawns treated with SMS or 20% AMLE showed a higher penetration at the end of the storage day when compared to the control. A lower value of penetration work is preferable as it indicates better texture of the *M. rosenbergii*.

## 4. Discussion

Recently, TPC and TFC of *A. muricata* leaves extraction were found to be comparatively higher than the present studies: 191.24 ± 0.03 mg GAE g^−1^ and 1777.47 ± 1.08 mg QE g^−1,^, respectively [34]. Similarly, Gyesi et al. 2019 [32] documented that the TPC of *A. muricata* leaves extraction was recorded at 4.38 ± 0.42 g GAE 100 g^−1^. Meanwhile, slightly lower amounts of TPC and TFC than the current studies were observed, which were recorded at 24.39 ± 0.001% and 21.49 ± 0.001% [30]. The value of TPC in *A. muricata* leaves depends on the origin of the plant, harvesting method, storage time, extraction method and the suitability of solvent used.

The catalytic activity of an enzyme in addition to substrate concentration is affected by the type and concentration of inhibitors [40]. Protease inhibition usually applies to the competition between two substrates in a reaction with an enzyme, where one can decrease the apparent rate of hydrolysis of the other. The AMLE contains various bioactive phytochemicals such as flavonoids, alkaloids, saponins, terpenoids and coumarins [41]. It also contains a high number of polyphenols which are capable of inhibiting protease activity. The binding of phenolic compounds to the protease enzyme disturbs the protein structure either by loosening or destabilizing the enzyme conformation and orientation of the substrate [42]. This is mostly expected in phenolic compounds with a bulky structure such as tannins and glycosides [42]. Polyphenols also become allosteric regulators and bind to the regulatory site of the enzyme, altering the conformational structure and causing deactivation [43].

Cathepsin B is a cysteine carboxypeptidase with the ability to cleave peptides into smaller tripeptides and dipeptides [44]. It is purified and identified from the hepatopancreas and muscle of *M. rosenbergii*. The AMLE could inhibit the cathepsin B activity by acting on the groove of the catalytic side cleft and binding to subsites from S1, S2, S1′ and S2′ of the enzyme [45]. The inhibition of cathepsin B by the AMLE could slow down the catalytic effect of the enzyme and, thereby, lead to the muscle softening of *M. rosenbergii* during storage.

Trypsin has been reported to be present in the hepatopancreas of *M. rosenbergii* and possesses collagenolytic activity in the prawn’s muscle [11]. *A. muricata* leaves extract contains caffeic acid, which inhibits trypsin through non-competitive inhibition and prevents trypsin from forming an enzyme–substrate complex [46,47]. However, complete inhibition of trypsin was not achieved at the tested range of AMLE concentrations. A similar result was reported by [48], where the tea polyphenols reduced the trypsin activity, but complete inhibition was not reported at the tested concentrations.

Collagen is the major protein content and plays a crucial role in the texture rigidity of the prawn [11]. The degradation of collagen could lead to the softening of muscle in *M. rosenbergii* [49]. Collagenase is an enzyme capable of hydrolyzing the native triple helix of collagen fibrils [10]. Therefore, it is important to inhibit the collagenase activity of the hepatopancreas of *M. rosenbergii* during post-harvest storage. In this instance, the inhibitory effects of the AMLE were studied against the collagenase activity of the crude protease extract from the hepatopancreas of *M. rosenbergii*. The inhibitory effect of the AMLE could be related to the presence of the phenolic compound in the extract [12]. Therefore, the treatment of *M. rosenbergii* with AMLE could retain the structural integrity of the muscle during storage by inhibiting collagenase activity from the hepatopancreas.

Firmness is defined as food material having high resistance to deformation by an applied force [50]. In this experiment, *M. rosenbergii* was subjected to a force by penetration test using a cylindrical probe at the second segment of the prawn. Firmness was determined as the maximum force applied to the prawn right before the probe penetrated the skin [51]. Firmness of the prawn is an important characteristic as it affects texture quality and freshness. High-quality prawns have a firm and juicy texture, whereas prawns with low quality have a mushy and soft texture [52]. Prawns with soft textures have low resistance to deformation by an applied force [50]. The low value of firmness corresponds to the high level of texture softening in *M. rosenbergii*.

The increasing value of firmness at earlier stages of storage could be explained by the initial muscle stiffness during rigor mortis. During rigor mortis, there is a glide between actin and the myosin filament due to adenosine triphosphate (ATP) decomposition [53]. The whole body of the prawn becomes inflexible and rigid [54]. The muscles of *M. rosenbergii* become hard, and more force is needed to deform the muscles. This results in an increase in firmness at an earlier stage of storage. As the storage time increases, proteases are released into the extracellular environment of the prawn. Proteases such as trypsin, cathepsin B and collagenase break down various protein components in the muscle structure [55]. There is an increase in the solubilization of specific myofibrillar proteins, especially the myosin heavy chain, in *M. rosenbergii* stored in ice [21]. The degradation of myofibrillar and sarcoplasmic proteins due to protease activity breaks down the muscle structure of *M. rosenbergii* [8]. The muscle loses its integrity and becomes soft [56]. The softening of muscle texture is represented by the decrease in firmness.

The high value of firmness is preferable as it indicates the better texture of *M. rosenbergii*. The highest firmness was observed in samples treated with SMS followed by AMLE (20%) at 8 days of storage. The high value of firmness could be explained by the ability of the AMLE to inhibit proteases, trypsin, cathepsin B and collagenase in *M. rosenbergii*. The activity of these enzymes was reported to progressively degrade actin and tropomyosin protein from the muscles of the prawns [57]. The activity of the hepatopancreatic proteases degraded perimysium and endomysium connective tissues as well as proteins localized in z-lines and h-zones [12]. The deterioration of protein in the muscle tissue by protease activity resulted in a soft texture and a decrease in firmness of *M. rosenbergii*. Treatment with the AMLE inhibited the enzymes. The inhibition of enzymes prevents or slows down the degradation of protein in the muscle tissue of *M. rosenbergii*. The integrity of the muscle of *M. rosenbergii* was maintained, and a high firmness value was achieved when compared to other treatment groups. Therefore, treatment of *M. rosenbergii* with 20% AMLE maintains firmness at up to 8 days of storage. The value was comparatively higher than firmness in the control group (*p* < 0.05).

An increasing value of penetration work indicated more work needed to penetrate the skin of *M. rosenbergii*. In the skin, there are two important proteins, which are elastin and fibrillin [58]. The proteins are responsible for skin elasticity and flexibility by forming an extensive elastic fiber network. Matrix metalloproteinase has the ability to cleave the elastin and fibrillin proteins [59]. In *M. rosenbergii*, matrix metalloproteinase, such as collagenase, actively degrades proteins during post-mortem storage [7]. The degradation of proteins breaks down the elastic fiber network and increases the stiffness and rigidity of the skin [60]. As storage time prolongs, the degradation of elastin and fibrillin by matrix metalloproteinases increases. This makes the skin of *M. rosenbergii* stiff and harder for the cylindrical probe to penetrate through; consequently, the penetration work of *M. rosenbergii* increases.

A high value of penetration work indicates low quality of *M. rosenbergii* due to the decreased elasticity of the prawn’s texture [61]. Due to the unwanted effect on texture, it is important to maintain a low value of penetration work in the prawn during post-mortem storage. The AMLE inhibitory effect against proteases, trypsin, cathepsin B and collagenase from the hepatopancreas prevents or slows down protein degradation in *M. rosenbergii*.

## 5. Conclusions

The AMLE at the concentration of 20% is able to inhibit the activity of proteases, trypsin and collagenase from *M. rosenbergii*. The dose-dependent inhibition of different protease activities was observed. The inhibitory effect of the AMLE was related to the presence of different total phenolic compounds in the extract. The phenolic compounds interact with the enzyme as an intermediate of the final product to inhibit enzyme activity. Meanwhile, SMS and 20% AMLE can maintain the firmness for up to 8 days of storage. A further study on the effectiveness of the AMLE coatings on sensory and psychochemical changes in *M. rosenbergii* has yet to be carried out.

## Data Availability

The data presented in this study are available on request from the corresponding author.
